# Premature Sperm Activation and Defective Spermatogenesis Caused by Loss of *spe-46* Function in *Caenorhabditis elegans*

**DOI:** 10.1371/journal.pone.0057266

**Published:** 2013-03-06

**Authors:** Wei-Siang Liau, Ubaydah Nasri, Daniel Elmatari, Jason Rothman, Craig W. LaMunyon

**Affiliations:** Department of Biological Science, California State Polytechnic University, Pomona, California, United States of America; Ecole Normale Superieure de Lyon, France

## Abstract

Given limited resources for motility, sperm cell activation must be precisely timed to ensure the greatest likelihood of fertilization. Like those of most species, the sperm of *C. elegans* become active only after encountering an external signaling molecule. Activation coincides with spermiogenesis, the final step in spermatogenesis, when the spherical spermatid undergoes wholesale reorganization to produce a pseudopod. Here, we describe a gene involved in sperm activation, *spe-46*. This gene was identified in a suppressor screen of *spe-27(it132ts),* a sperm-expressed gene whose product functions in the transduction of the spermatid activation signal. While *spe-27(it132ts)* worms are sterile at 25°C, the *spe-46(hc197)I; spe-27(it132ts)IV* double mutants regain partial fertility. Single nucleotide polymorphism mapping, whole genome sequencing, and transformation rescue were employed to identify the *spe-46* coding sequence. It encodes a protein with seven predicted transmembrane domains but with no other predicted functional domains or homology outside of nematodes. Expression is limited to spermatogenic tissue, and a transcriptional GFP fusion shows expression corresponds with the onset of the pachytene stage of meiosis. The *spe-46(hc197)* mutation bypasses the need for the activation signal; mutant sperm activate prematurely without an activation signal in males, and mutant males are sterile. In an otherwise wild-type genome, the *spe-46(hc197)* mutation induces a sperm defective phenotype. In addition to premature activation, *spe-46(hc197)* sperm exhibit numerous defects including aneuploidy, vacuolization, protruding spikes, and precocious fusion of membranous organelles. Hemizygous worms [*spe-46(hc197)/mnDf111*] are effectively sterile. Thus, *spe-46* appears to be involved in the regulation of spermatid activation during spermiogenesis, with the null phenotype being an absence of functional sperm and hypomorphic phenotypes being premature spermatid activation and numerous sperm cell defects.

## Introduction

Sperm activation is an event that requires precise timing. Precocious activation may either result in premature consumption of stored energy or interfere with efficient fertilization. Belated activation may allow competing sperm to gain the advantage in the race to fertilize the ova. For many species, activation coincides with sperm release from the male. For instance, sea urchin sperm activate upon release into water column due to an influx of Na^+^ from the seawater [1]. The resulting spike in intracellular pH stimulates motility and respiration through a signal transduction pathway that includes Ca^2+^, cAMP, and cAMP dependent kinases. The outcome is the activation of dynein ATPase in the flagellum and increased mitochondrial activity [2]–[4]. Thus, sea urchin sperm activate precisely when motility is critical, and the cue is from the external environment.

For other species, the cue to activate is a signaling molecule. In mammals, bicarbonate is the activation cue. Bicarbonate not only induces a Ca^2+^ influx, cAMP accumulation, and cAMP dependent protein kinase A activity, but it also increased motility and respiration [5]–[8]. As a component of the seminal fluid, bicarbonate stimulates sperm activation precisely when the sperm enter the female reproductive tract and require motility to reach the ova and compete with rival sperm. The similarity between sea urchin and mammalian sperm activation suggests that the cellular processes are conserved across distantly related taxa.

In the nematode *Caenorhabditis elegans*, sperm activation occurs during the final step in sperm development, spermiogenesis. During activation, the spherical spermatids undergo wholesale cytoplasmic reorganization to form a pseudopod, which propels the sperm to the fertilization site. In *C. elegans,* sperm are produced by both males and hermaphrodites, each type activating at its own precise time point: (i) male sperm at ejaculation, and (ii) hermaphrodite sperm at the time they arrive at the spermatheca. Only recently has an activation signaling molecule been identified. The male spermatids activate within the uterus when exposed to seminal fluid containing the protease TRY-5 [9]. The signaling molecule for hermaphrodite sperm remains elusive. Intracellularly, the events of *C. elegans* spermatid activation include an influx of cations [10], a brief elevation in pH [11], the release of intracellular Ca^2+^ [12]–[14], polymerization of MSP, and fusion of the membranous organelles (MOs) with the plasmalemma [12].

While an external signal induces activation in *C. elegans* sperm, precise control over activation is brought about antagonistically: a set of inhibitory, or “brake,” proteins maintains the spermatid stage until the activation signal relieves the inhibition [15]–[16]. In hermaphrodites, the signaling cascade for activation involves a set of proteins known as the “SPE-8 group”: SPE-8 [17], SPE-12 [18]–[19], SPE-19 [20], SPE-27 [21], and SPE-29 [22]. Male *spe-8* group mutants are fertile, but mutant hermaphrodites are self-sterile because their spermatids fail to activate. Notably, the arrested hermaphrodite self-spermatids can be “rescued” when exposed to seminal fluid from males, which, along with other data, suggests that SPE-8 group proteins serve specifically to amplify the activation signal for hermaphrodite self sperm [12] [19].

The brake proteins that prevent activation include SPE-6 [15] and SPE-4 [16]. These proteins have functions earlier in spermatogenesis that center on the fibrous body-membranous organelle complexes (FB-MOs). The FB-MOs act to shuttle the Major Sperm Protein (MSP) through the meiotic divisions. The most abundant protein in sperm, MSP is encoded by numerous paralogs [23], is packaged into the FBs in the primary spermatocyte and released into the cytosol of the spermatid [24]. MSP is polymerized into fibers in the pseudopod of the spermatozoon [25] thereby driving motility through treadmilling of the pseudopod membrane [26]. The SPE-4 protein localizes to the MO membrane, and null *spe-4* mutant sperm have fibrous bodies (FBs) that fail to associate normally with the membranous organelles (MOs) to form normal FB-MO complexes [27]–[28]. Null *spe-6* mutants fail to package the Major Sperm Protein (MSP) into the FBs [29]. However, at a later developmental stage, current models suggest that SPE-4 and SPE-6 function as brake proteins prior to entry into spermiogenesis [15]–[16]. Certain hypomorphic mutations in *spe-4 and spe-6* suppress the sterility of *spe-27(it132)* mutants. These mutations were recovered in a suppressor screen of *spe-27(it132)* [17]. These *spe-27* suppressor mutations not only restore fertility to other *spe-8* group mutants, but they also result in premature spermatid activation. Here, we report the identification of *spe-46*, another gene isolated in the suppressor screen of *spe-27(it132).* The *spe-46(hc197)* mutation causes premature spermatid activation, and results in improper segregation of nuclei during meiotic divisions.

## Results and Discussion

### Identification of *spe-46* Mutation, *hc197*

**Figure 1:**
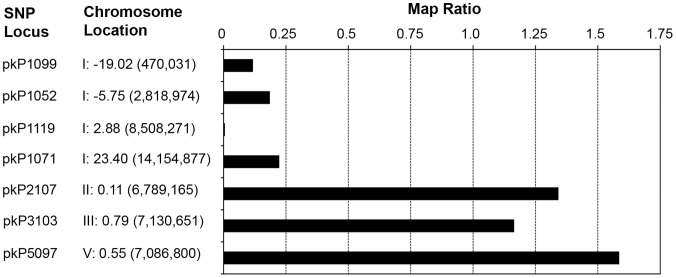
SNP mapping data for *hc197* on Chromosome I. Map ratios near zero indicate linkage, while ratios near 1 indicate independent segregation. The SNP loci and chromosome locations (chromosome number, genetic map position, physical map location) are indicated.

To identify the *hc197* mutation, we used whole genome sequencing, which required only a rough estimate of the chromosomal location. Because *hc197* segregated independently from the Chromosome IV marker, *unc-22,* we knew it was on another chromosome. We also ruled out the X chromosome since no sperm-expressed genes are known to reside there [30]. Single nucleotide polymorphism (SNP) mapping as described by Wicks et al. [31] was used to identify the approximate chromosomal location of *hc197*. Briefly, after crossing our *hc197* mutant strain to the polymorphic Hawaiian strain CB4856, pooled mutant and non-mutant F2 were subjected to PCR/RFLP analysis, and map ratios were calculated for the SNP loci examined. The map ratios near 1.0 for SNPs in the middle of Chromosomes II, III, and V indicate *hc197* is located elsewhere (Fig. 1). We initially analyzed pkP1119 on Chromosome I and got a map ratio near zero, indicating the SNP was linked to *hc197.* Our initial results were confirmed by very small map ratios from three more SNP loci across Chromosome I (Fig. 1), but pkP1119 had the smallest map ratio, suggesting it was closest to *hc197*.

We proceeded to sequence the genome of the *hc197; spe-27(it132) unc-22(e66)* strain and obtained a read depth of 62 reads per base pair. The entirety of Chromosome I was examined for SNPs that created non-synonymous alterations to coding sequences and that were present in greater than 80% of the reads. At the same time the *hc197* mutant strain was sequenced, we also sequenced the genomes of six other *spe-27* suppressor mutants isolated in the same screen that produced *hc197.* The SNPs found in *hc197* were compared to those identified in the six other mutant genomes. Shared mutations were omitted from consideration, as they could not be the unique mutation responsible for the *hc197* suppressor phenotypes. The remaining nine novel mutations are shown in Table 1. All nine mutations were common transition mutations induced by ethyl methanesulfonate (EMS).

**Table 1:** Novel^1^ non-synonymous mutations on Chromosome I.

Nucleotide Position	AffectedCDS	Genetic location	Mutationtype	AA^6^change	*fem-3/fem-1* 2	Expression^3^	Phenotype^4^
7678275	*T24B1.1*	2.23	C to T	H to Y	–	Hermaphrodite; Sex enriched	–
9057658	*W06D4.2*	3.45	C to T	Stop	35.75	Sperm enriched	–
9142094	*ZK858.7*	3.63	C to T	G to E	0.28	Protein expression	Multiple somatic
9635933	*ppfr-1*	3.76	C to T	A to T	–	Embryonic	Oocyte meiotic chromosome segregation^5^
9655212	*T05F1.11*	3.83	C to T	H to Y	0.95	Protein expression	Multiple somatic
9689655	*rpl-14*	3.85	C to T	S to F	0.88	Multiple somatic	Multiple somatic
11393497	*aat-9*	7.96	C to T	A to T	0.54	Germline; Oocyte; Nervous system	–
11540790	*dys-1*	9.05	C to T	G to R	0.89	Muscle; Nervous system	Movement defects
12905311	*car-1*	14.18	G to A	G to R	0.27	Oogenesis; Apoptosis	Germline cell death; Oocyte defects

1 We sequenced two other mutant genomes from the same mutation screen. Mutations in common were not considered.

2 Microarray data comparing sperm enriched (*fem-3*) to oocyte enriched (*fem-1*) mRNA [44] [30].

3 Data from microarray studies as reported on Wormbase [52].

4 Data from mutant and RNAi studies as reported on Wormbase [52].

5 See Han, 2009 [53].

6 AA: amino acid.

Because the *hc197* mutation suppresses the phenotype of a sperm-expressed gene, it should be expressed in sperm and have a sperm defective, or sterile, phenotype. Wormbase (www.Wormbase.org) contains a comprehensive collection of such data for *C. elegans*. In Table 1, the phenotype and expression pattern for each gene identified by Illumina sequencing are presented. Phenotypic data come primarily from RNAi experiments, which have been a powerful resource for rapid gene knockdown [32], although RNAi is ineffective for sperm-expressed genes [33]. Therefore, RNAi experiments should not produce a sperm defective phenotype, but significant non-sperm phenotypes suggest expression partially or solely in somatic tissues. None of the genes presented had a sperm defective phenotype, but many did have somatic phenotypes. The Wormbase expression patterns come primarily from multiple microarray experiments, and we paid close attention to expression patterns related to sperm or the germline. Two genes are involved in oogenesis (*aat-9* and *car-1*), but both also function in non-germline tissues [34]–[35]. Two other genes, T24B1.1 and W06D4.2, were of particular interest. T24B1.1 was annotated as sex-enriched, and W06D4.2 had a sperm-enriched expression pattern. Finally, we examined microarray data comparing transcript abundance in hermaphrodites making only sperm [*fem-3(q23)*] with hermaphrodites making only oocytes [*fem-1(hc17)*] [30]. Only W06D4.2 had a ratio indicating sperm expression, although the fem-3/fem-1 ratio for T24B1.1 was not available. The mutations identified in both genes were further confirmed by conventional sequencing. Because both W06D4.2 and T24B1.1 are located near pkP1119, the presumptive locale for *hc197*, we decided to pursue both genes.

**Figure 2:**
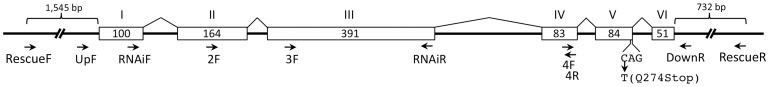
The gene structure of *W06D4.2.* Boxes represent individual exons (I-VI). The *hc197* mutation (C −>T) converts a glutamine residue to a premature stop. Horizontal arrows refer to oligonucleotide sequences. RescueF and RescueR were used to amplify the rescuing 3,626 bp fragment. RNAiF and RNAiR provided the RT-PCR amplicon (585 bp) that was used to construct an RNAi feeding clone. The UpF and DownR primers were used to amplify the cDNA sequence, which was confirmed by conventional DNA sequencing employing the remaining primers.

To determine the gene that harbors the *hc197* mutation, we performed transformation rescue. PCR amplified wild-type products of both W06D4.2 and T24B1.1 including ∼1Kbp of promoter and ∼1Kbp of downstream sequence were injected into the gonads of hermaphrodites with the *hc197* mutation but an otherwise wild-type background. F2 transformants were reared at 25°C from the L4 stage onward and examined for rescue of the fertility defect associated with *hc197*. Transformants with wild-type T24B1.1 had the same fertility as did control worms, so T24B1.1 failed to rescue the mutant phenotype (Table 2). However, W06D4.2 did result in rescue: transgenic F2 were significantly more fertile than were controls (Table 2). These results confirm that the *hc197* mutation resides in W06D4.2, which is henceforth referred to as *spe-46.* We confirmed the exonic arrangement of *spe-46* by sequencing the cDNA derived from RT-PCR. Based upon EST sequences mapping to *spe-46* in Wormbase, there is no alternative splicing for this gene. The mutation changes the last codon in the fifth exon from a glutamine to a stop (Fig. 2).

**Table 2:** Results from transformation rescue experiments.

	*T24B1.1* *			*W06D4.2* **		
	Transgenic F2	Control F2	Statistical Result	Transgenic F2	Control F2	Statistical Result
Mean No. Progeny (N)	22.0 (14) ±2.96	23.4 (13) ±3.91	t = −0.30 P = 0.7661	210.2 (16) ±13.15	42.6 (15) ±3.05	t = 12.431 P = 8×10^−10^

* *hc197; him-5(e1490)* worms were transformed with *T24B1.1.*

** *hc197* worms with an otherwise wildtype genomic background were transformed with *W06D4.2.*

Sample Sizes are Listed in Parentheses, and Error is Shown as ± SEM

### *spe-46* Encodes a Predicted Membrane Protein with Sperm-specific Expression

**Figure 3:**
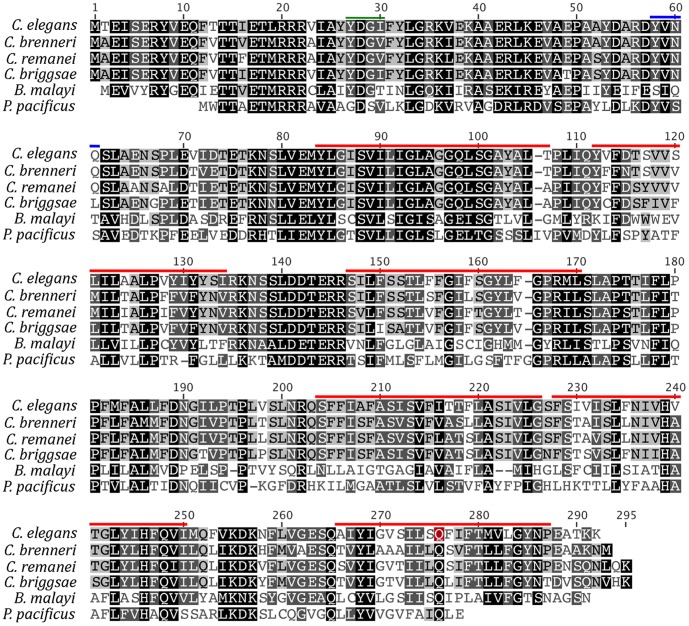
An alignment of the *C. elegans* SPE-46 protein sequence with those identified as homologs in other nematode species. The Geneious Alignment option with Blosum62 cost matrix generated the alignment. Red bars represent predicted transmembrane domains. The green bar represents a predicted Tyr-based sorting signal, and the blue bar represents both a predicted Tyr phosphorylation site and a predicted SH2 binding domain. The Q residue highlighted in red is transformed into a stop codon by the *hc197* mutation.

Sequence analysis with the ELM resource [36] suggests that the SPE-46 protein has six putative transmembrane domains and a Tyr-based sorting signal important in trafficking proteins to cellular compartments [37] (Fig. 3). Interestingly, both the ELM resource and the NetPhos 2.0 Server [38] predict a Tyr phosphorylation site at the same position the ELM predicts an SH2 binding domain (AA position 58–61; Fig. 3). Hypothetically, this site could interact with SPE-8 protein, a kinase with an SH2 domain and which transduces the signal to activate [17]. The NetPhos 2.0 Server also predicts numerous other Ser and Thr phosphorylation sites on the SPE-46. The premature stop caused by *hc197* falls within the final transmembrane domain. SPE-46 appears exclusive to nematodes, as homologs were not found in other taxa, although the nematode sequences are highly conserved (Fig. 3).

**Figure 4:**
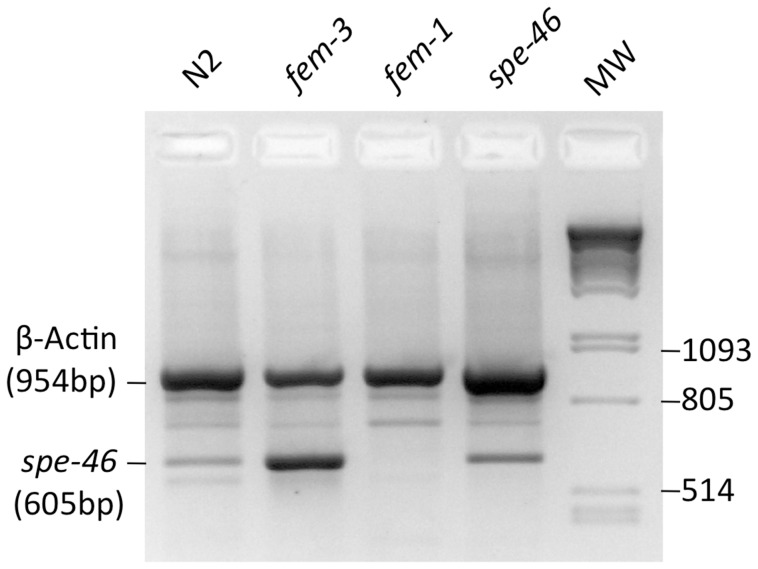
Products of RT-PCR reactions using RNA from wild-type (N2) worms, hermaphrodites that produce only sperm [*fem-3(q23)*], hermaphrodties that produce only oocytes [*fem-1(hc17)*], and *spe-46(hc197)* mutant hermaphrodites. The reactions were multiplexed with primers for both the *spe-46* transcript and the transcript for the *C. elegans* β-Actin homolog *act-2*. The molecular weight marker (MW) is lambda phage cut with PstI. While non-specific products were amplified, the specific amplicons for both *spe-46* and *act-2* were present. In particular, the *spe-46* product is present in all lanes but *fem-1*, a strain that makes no sperm. The size of each specific amplicon is indicated.

While microarray studies suggest that *spe-46* is highly sperm-specific, we sought to test this conclusion by RT-PCR. Our results show that the *spe-46* transcript is only present in animals making sperm cells [*fem-3(q23)* mutants] and not in animals making only oocytes [*fem-1(hc17)* mutants] (Fig. 4). The *spe-46* product was amplified using primers 2F and 4R (Fig. 2), and the expression in *spe-46* mutants was similar to that from wild-type worms, suggesting that the transcripts with the premature stop codon appear stable.

**Figure 5:**
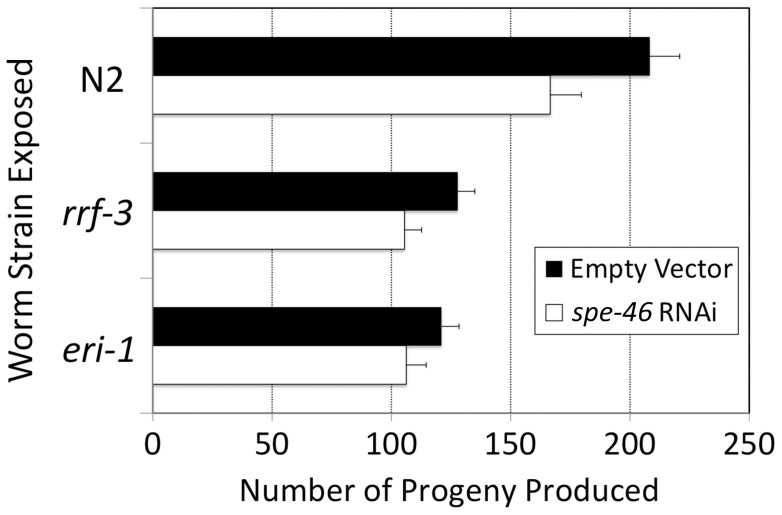
The effect of *spe-46* RNAi on lifetime fecundity. Worms were exposed to transformed *E. coli* strain HT115 expressing dsRNA corresponding to the *spe-46* transcript (*spe-46* RNAi) or to control bacteria transformed with only the RNAi vector, L4440 (empty vector). In addition to wild-type strain N2, the strains *rrf-3* and *eri-1* were utilized due to their increased sensitivity to RNAi. Error bars represent the SEM.

The sperm specificity of *spe-46* expression was also tested by looking for somatic defects in response to RNA interference (RNAi) for *spe-46*. We constructed an RNAi feeding strain of *E. coli* HT115 expressing double stranded RNA corresponding to 585 bp of the cDNA (amplified with primers RNAiF and RNAiR – see Fig. 2). In addition to wild type strain N2, the RNAi sensitive strains *rrf-3(pk1426)II* and *eri-1(mg366)IV* were exposed to *spe-46* RNAi. In no case did we observe evidence of somatic phenotypic defects such as dumpy, uncoordinated, slow growth, etc. The exposed worms appeared somatically normal, especially compared to control worms exposed to bacteria containing the RNAi empty vector. The only difference was that RNAi exposed worms produced slightly but significantly fewer progeny than did controls (Fig. 5; *F_1.77_* = 10.554; *P* = 0.002). Such a result cannot necessarily be interpreted as a *spe-46* specific sperm defect because sperm-expressed genes in *C. elegans* are typically resistant to RNAi [33]. Alternatively, it is possible that the reduction in fertility is due to a non-specific effect of titrating away limited component(s) of the RNAi machinery, which are active during spermatogenesis [39].

**Figure 6:**
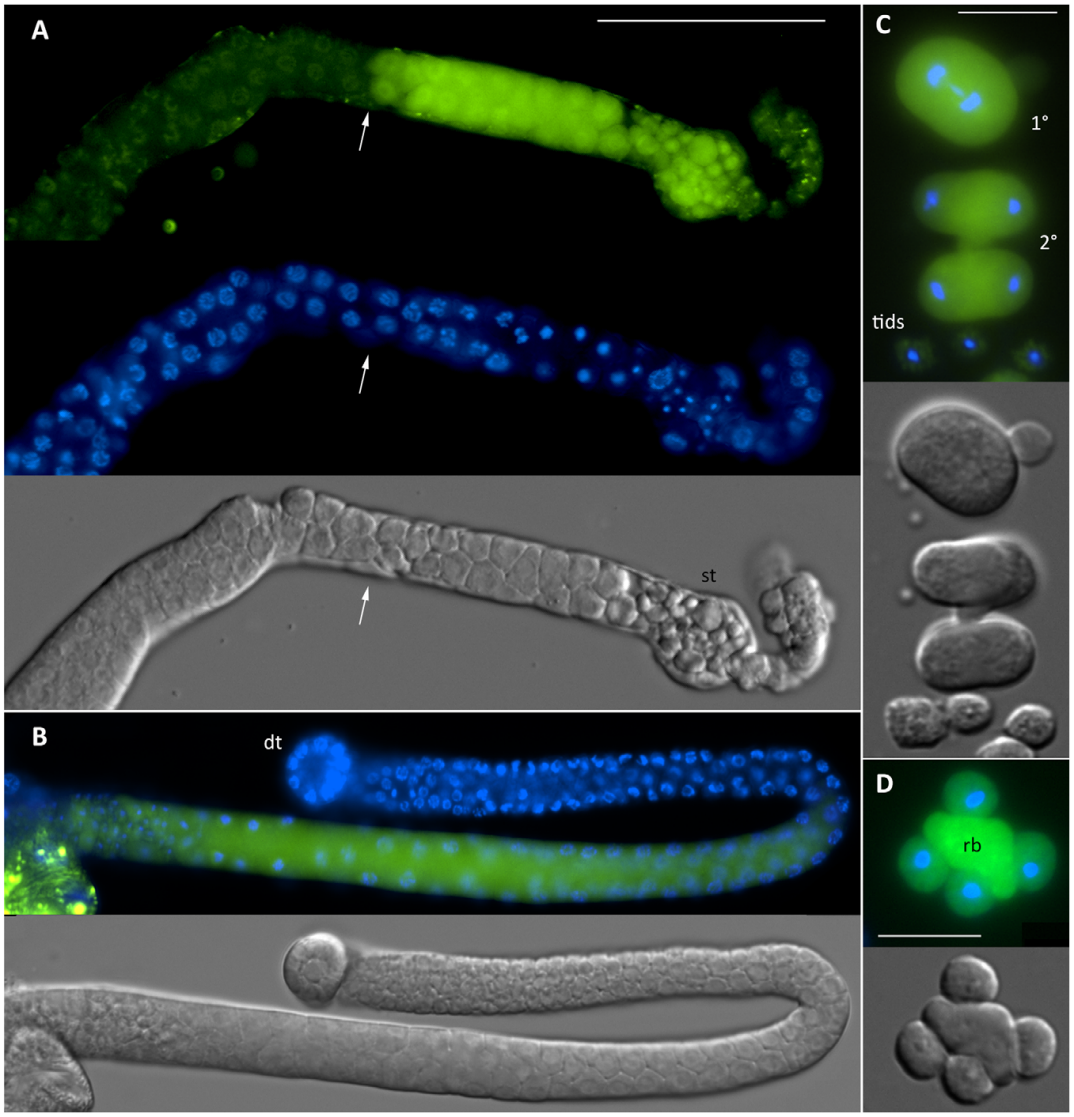
Expression of the *spe-46:GFP* reporter. Here, the worms expressed an integrated GFP flanked by the *spe-46* promoter and 3′ UTR, and the tissue was labeled with the DNA dye Hoechst 33342. (A) Fluorescence was found in a specific set of cells in the gonads of L4 hermaphrodites, likely those that will produce sperm. The arrow provides reference for the region where GFP expression begins. Gamete maturation proceeds to the right, with the sperm taking up residence in the spermatheca (st). (B) In male gonads, GFP expression corresponds with the transition zone and entry into meiotic pachytene (approximately at the bend in the gonad). dt refers to the distal tip of the gonad. (C) The primary (1°) and secondary (2°) spermatocytes, and spermatids (tids) are shown. (D) The GFP was detected in the residual body (rb) during the second meiotic division. The scale bar in A is 50 µm and is the same reference for B. The bars in C and D represent 10 µm.

The expression of SPE-46 was investigated using a transcriptional GFP fusion. Our reporter construct included 1 Kbp of genomic sequence on either side of the *spe-46* coding sequence with GFP replacing the *spe-46* coding sequence. Green fluorescence was localized to the spermatogenic tissue (Fig. 6). In gonads from hermaphrodite L4 larvae, GFP is present in a very specific population of cells, likely those that will produce the sperm (Fig. 6A). In the male gonad, GFP labeling begins at approximately mid to late pachytene stage, and it is present through the appearance of the sperm (Fig. 6B). Both primary and secondary spermatocytes contain GFP, as do the sperm (Fig. 6C, D). Unfortunately, we were unsuccessful in out attempts to determine the subcellular localization of SPE-46 via construction of a translational GFP fusion. We are left to conclude that *spe-46* expression occurs in the spermatogenic tissue beginning with entry into the meiotic pachytene phase.

### *spe-46(hc197)* is a Recessive Suppressor of the Spermiogenesis Activation Pathway and has a Conditional Spermatogenesis-defective Phenotype on its Own

**Figure 7:**
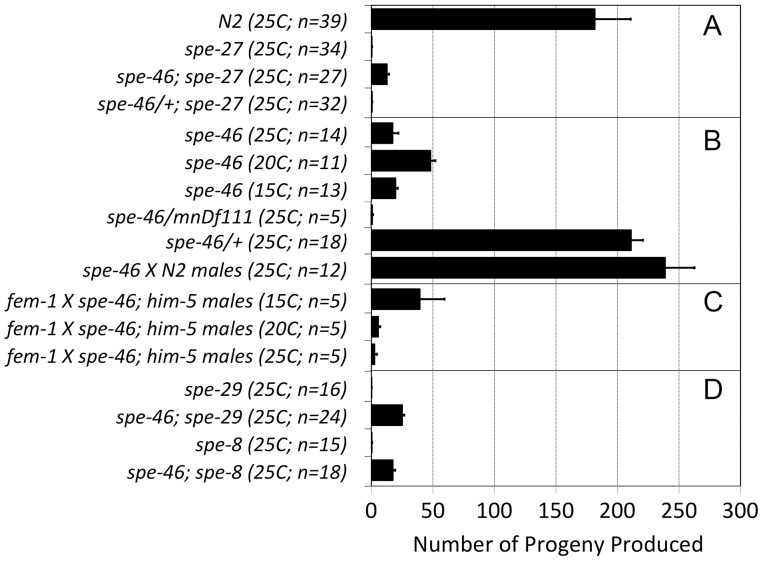
The number of progeny associated with the *spe-46(hc197)* mutation. (A) Hermaphrodite self-progeny showing that *spe-46(hc197)* is a recessive suppressor of *spe-27(it132)* sterility. (B) In an otherwise wild-type background, selfing *spe-46* hermaphrodites exhibit a conditional spermatogenesis defect that is rescued by mating with N2 males. Further, the *spe-46* defect is recessive. (C) Male *spe-46* mutant worms display a temperature-sensitive sperm defect when mating with spermless *fem-1(hc17ts)* hermaphrodites, being most fertile at 15°C. Finally, (D) the *spe-46(hc197)* mutation suppresses mutations in both *spe-8* and in *spe-29,* restoring partial hermaphrodite self fertility. Error bars represent SEM.

Worms homozygous for *spe-27(it132ts)* are sterile at 25°C, unless they are also homozygous for *spe-46(hc197)* (Fig. 7). This suppression of *spe-27* sterility is a recessive phenotype, as *spe-46(hc197)/+* are sterile in a *spe-27* background. It was previously shown that the *spe-4(hc196)* and *spe-6(hc163)* suppressor alleles restore fertility not only to *spe-*27 mutants but also to mutants of the other *spe-*8 group genes [15]–[16]. Given this fact, we tested the ability of *spe-46(hc197)* to suppress the sterility of mutations in the spermiogenesis genes *spe-8,* and *spe-29.* In both cases, *spe-46(hc197)* restored fertility to the other spermiogenesis gene mutants (Fig. 7). Therefore, the suppression phenotype of *spe-46(hc197)* is neither allele specific nor gene specific, suggesting that it bypasses the spermiogenesis pathway in a manner similar to *spe-4* and *spe-6* suppressor alleles.

Further evidence supporting a bypass hypothesis is that *spe-46(hc197)* causes premature spermatid activation. Virgin male *him-5(e1490)* worms contained only inactive spermatids when dissected into SM1 buffer, whereas nearly 30 percent of the sperm within *spe-46(hc197); him-5(e1490)* mutant males were active spermatozoa under the same conditions (see below). The existence of spermatozoa within virgin males shows that they activated prematurely, and they are likely the source of male infertility because crawling spermatozoa obstruct sperm transfer during copulation [40]. These results suggest that the SPE-46 protein inhibits spermiogenesis until the activation signal is received by the spermatid. Including SPE-4 [16] and SPE-6 [15], the addition of SPE-46 makes three proteins that inhibit spermiogenesis.

In an otherwise wild-type background, *spe-46(hc197)* worms have reduced fertility that is influenced by temperature: they produce more progeny at 20°C than they do at 25°C or 15°C. This fertility deficit is due to a sperm defect, because the fertility of *spe-46(hc197)* hermaphrodites increases after wild type males supply sperm at mating (Fig. 7). Furthermore, the *spe-46(hc197)* phenotype is recessive; *spe-46(hc197)/+* worms have wild-type fertility. The fertility deficit is also evidenced in males. When paired with spermless *fem-1(hc17)* hermaphrodites at 25°C, male *spe-46(hc197); him-5(e1490)* sired very few offspring (Fig. 7), but the number of cross progeny increased with lowered temperature (Fig. 7), indicating that the male phenotype is also temperature sensitive. To understand the nature of the *spe-46(hc197)* mutation, we placed it over the *mnDf111*, a large deficiency that removes *spe-46* entirely. The hemizygous hermaphrodites were essentially self-sterile (Fig. 7; control worms with wild-type *spe-46/mnDf111* were fertile), suggesting that the null phenotype of *spe-46* is complete sterility.

**Figure 8:**
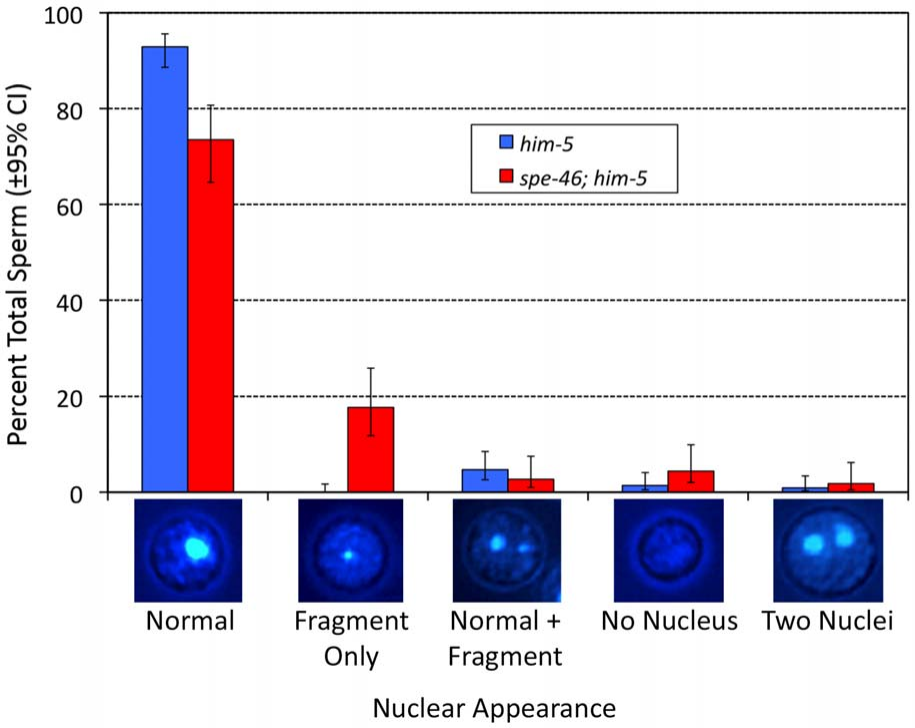
The appearance of nuclei in spermatids labeled with Hoechst 33342. Sperm were observed under epifluorescence, and multiple images were taken at various focal planes to ensure all nuclei were observed. The focal planes were combined to produce the images below the graph. The sperm phenotypes are expressed as percent total sperm, and the confidence interval (CI) is indicated.

While much of the self-fertility defect in homozygous *spe-46(hc197)* hermaphrodites is due to a lack of fertilization, we also found a defect in embryogenesis, but only at 25°C. Of 89 fertilized eggs laid by *spe-46(hc197)* hermaphrodites at 25°C, only 41 hatched, giving an embryonic failure rate of 53.9%. The embryonic failure rate was 0% at 20°C (of 82 eggs) and 1.6% at 15°C (of 125 eggs). Every hatchling at all three temperatures survived to adulthood. This embryonic failure rate at 25°C was rescued in transgenic worms: all 128 eggs laid were fertilized, and 116 hatched, for a failure rate of only 9.4%. Given the sperm specificity of *spe-46* expression, the observed embryonic failure is likely due to sperm that fertilized oocytes but were incapable of producing viable embryos. We found that nearly 20% of the sperm from *spe-46(hc197); him-5(e1490)* males appeared to have only a small fragment of the normal sperm nucleus (Fig. 8). This aneuploidy defect was almost never observed in control *him-5(e1490)* male sperm (Fig. 8), so we attribute this defect to *spe-46(hc197),* although we cannot rule out a synergistic effect between *spe-46* and *him-5*. Other defects, including sperm with no nucleus, two nuclei, and a normal nucleus plus a fragment, were observed at very low levels in sperm from both strains (Fig. 8) Sadler and Shakes [41] showed that even anucleate sperm can fertilize oocytes and initiate early embryo development. We suspect that some nuclei that appeared normal were actually aneuploid, having excess numbers of chromosomes that were all packaged together. It is likely that self-sperm from *spe-46(hc197)* hermaphrodites are similarly afflicted by aneuploidy, since SPE-46 is expressed in both male and hermaphrodite spermatogenesis. While our data cannot rule out mitotic chromosomal non-disjunction, it is likely that aneuploidy occurs as a result of meiotic non-disjunction, given that our GFP reporter is expressed during meiosis and not earlier during the mitotic division (Fig. 6). These results lead to the conclusion that the SPE-46 protein participates in the meiotic divisions, in addition to its role in maintaining the spermatid stage. Such multiple roles are not unexpected, given that both SPE-4 and SPE-6 have roles early in sperm development and later in spermiogenesis inhibition [15]–[16].

**Figure 9:**
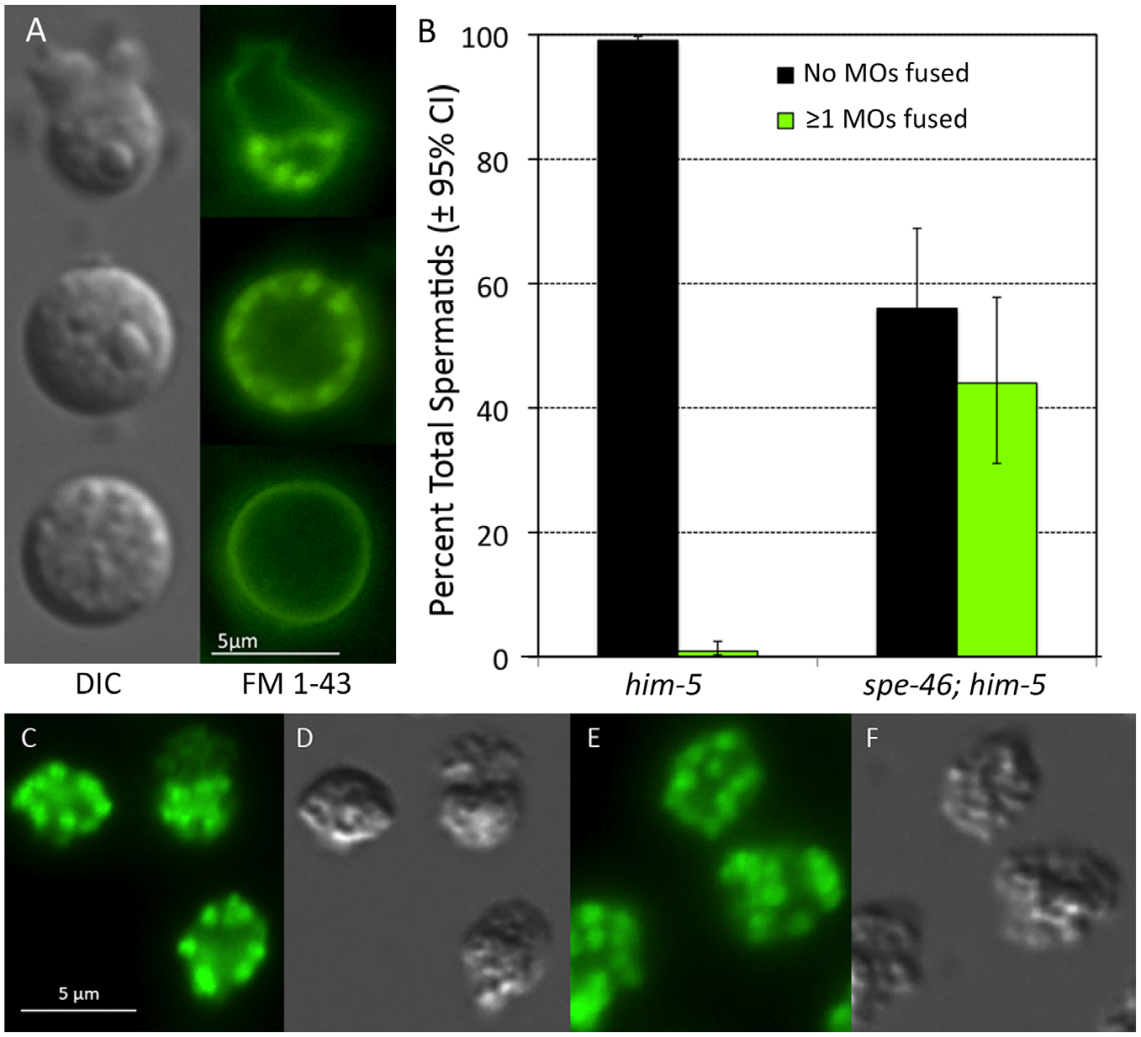
The status of the membranous organelles (MOs) in sperm. A) When labeled with the membrane dye FM® 1–43 (Life Technologies™), fused MOs are visible as bright spots just inside the cell membrane, but unfused MOs are not labeled. Here, the sperm are shown in both DIC illumination and epifluorescence illumination. Top panel: a spermatozoon with an obvious pseudopod and the bright foci in the cell body indicating fused MOs. Middle panel: an abnormal spermatid with fused MOs. Bottom panel: a normal spermatid with no fused MOs. B) The percentage of normal spermatids and those that had at least a single obvious fused MO from *him-5* and *spe-46; him-5* male worms. Error bars represent SEM. In C-F, the sperm have been fixed, permeabilized, and exposed to an Alexa Fluor® 594 conjugate of wheat germ agglutinin (WGA; Life Technologies™), which labels all MOs, including those that have not fused with the cell membrane (original red fluorescence false colored green). C) Sperm from a *spe-46(hc197); him-5(e1490)* male. D) The same cells as is C, visualized in DIC optics. E) Sperm from a *him-5(e1490)* male labeled as in C. F) DIC image of the cells in E.

Furthermore, mutant males harbored spermatids with numerous defects. First, a large fraction of spermatids had prematurely fused MOs (Fig. 9A). Normally, the MOs fuse with the cell membrane only during spermatid activation, creating a pore in the cell membrane, which becomes contiguous with the interior of the MO. The lipid dye FM®1–43 (Life Technologies™) labels the cell membrane, and if any MOs have fused, they become labeled by FM®1–43 as well resulting in fluorescent foci abutting the cell membrane [14]. When exposed to FM®1–43, 44% of spermatids from *spe-46(hc197); him-5(e1490)* males had fused MOs, yet these cells showed no sign of activating (Fig. 9B). Only rarely did spermatids from *him-5(e1490)* have anything resembling a fused MO (Fig. 9A). Even though many MOs fused abnormally in *spe-46(hc197); him-5(e1490)* sperm, they segregated normally during the post-meiotic budding division. We examined >100 sperm from mutant males, and they contained a normal complement of MOs in a visual comparison to those from *him-5(e1490)* males (Fig. 9C-F). Therefore, *spe-46(hc197)* sperm have normal a number of MOs, but many of those MOs fuse prematurely with the cell membrane.

**Figure 10:**
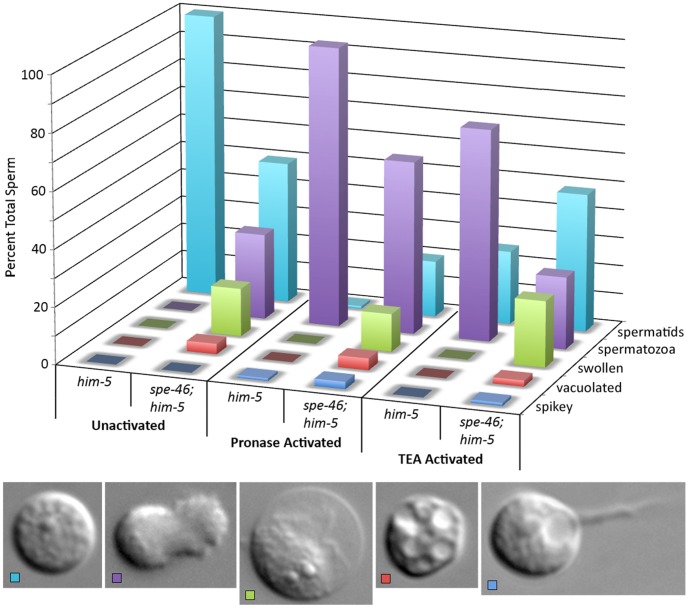
The sperm phenotypes from *spe-46(hc197); him-5(e1490)* males. Males were dissected in SM1 buffer (unactivated), in SM1 plus Pronase (200 µg/ml) (pronase activated), and in SM1 plus 70 mM Triethanolamine (TEA) (TEA activated). Sperm phenotypes are categorized into: (i) spermatid (blue), (ii) spermatozoa (purple), (iii) sperm that appeared swollen (green), (iv) sperm that had large vacuoles (red), and (v) sperm with extended spikes (blue). The number is expressed as percent total sperm. DIC image of sperm phenotypes are shown at the bottom of the graph. Scale bar is 5 µm.

Many other morphological defects were obvious in mutant sperm. Some had large vacuoles, while others had prominent protruding spikes, and yet others were swollen (Fig. 10). The vacuolated and swollen defects were never observed in *him-5(e1490)* male sperm under identical conditions (Fig. 10). Swollen sperm likely had been active spermatozoa; they typically had fused MOs, and normal sperm activated *in vitro* sometimes take on the swollen phenotype after an extended period (CWL, personal observations).

We also examined the ability of the sperm to activate in response to *in-vitro* activating chemicals [11]. Sperm from *him-5(e1490)* males behaved normally, with 98% activating to spermatozoa after exposure to Pronase (Fig. 10). Sperm from *spe-46(hc197); him-5(e1490)* did not respond so completely to Pronase, with nearly 20% remaining as spermatids (Fig. 10). Exposure to triethanolamine (TEA) caused more than 75% of sperm from *him-5(e1490)* males to activate, but *spe-46(hc197); him-5(e1490)* sperm did not appear to respond, with nearly identical proportions of spermatids and spermatozoa compared to sperm exposed just to SM1 buffer (Fig. 10). These results demonstrate that some spermatids from *spe-46(hc197)* mutant males are incapable of activating in response to *in-vitro* activators, suggesting that they cannot activate *in-vivo*.

### Conclusions

Our results support the idea that spermatid activation requires proper timing. Spermatids from *spe-46(hc197)* worms activate prematurely to spermatozoa, and mutant males exhibit a near complete loss of fertility. Prematurely activated spermatozoa are known to compromise male fertility as they obstruct passage of sperm from male to hermaphrodite at mating [9]. Premature activation associated with the *spe-46(hc197)* mutation also bypasses the need for an activation signal and its transduction through the *spe-8* group gene products. Not only is a fraction of the sperm prematurely activated, a large portion of the inactive spermatids have prematurely fused MOs. These results support the hypothesis that SPE-46 acts as a brake protein to inhibit spermiogenesis in the manner of SPE-4 and SPE-6 [15]–[16]. Under this hypothesis, SPE-46 has a role in preventing spermiogenesis until it is down-regulated by an incoming activation signal or by the *hc197* mutation. We also show that SPE-46 expression begins when the developing gamete nuclei reach the late pachytene stage, and that SPE-46 has a role in the meiotic process. These two roles are evidenced by the degree of loss of SPE-46 function: worms with two mutated alleles exhibit a host of defects including premature spermatid activation, while hemizygous worms which have only a single mutated allele are without competent sperm altogether.

SPE-46 is predicted to reside in a membrane, but its subcellular location is as yet undetermined. The *spe-4* and *spe-6* genes, both of which can be mutated to cause premature spermatid activation, also have functions earlier in spermatogenesis associated with assembly of the FB-MO complex. Mutations in both genes affect FB-MO function [27] [29], and the SPE-4 protein is located in the MO membrane [27]. Recently, *spe-44* has been identified as a master transcriptional regulator of a large subset of sperm-expressed genes, but it does not directly regulate expression of *spe-4, spe-6,* or *spe-46* [42]. Given that SPE-4 and SPE-6 are associated with the MO, it is a likely destination for the SPE-*46* protein. However, given the premature fusion of MOs (Fig. 8), the cell membrane is also a possible location. Resolution of this question is the subject of our future investigations.

SPE-46 is highly conserved in nematodes, but it is not present in other organisms. Given the intense scrutiny of Chromosome I sperm-expressed genes via traditional mutagenesis [43], it is surprising to discover new Chromosome I sperm-expressed genes by mutagenesis. However, we feel that the *spe-27(it132)* suppressor screen that identified *spe-46* provides an increased level of sensitivity for the identification of genes involved in spermiogenesis. We have at least two more *spe-27(it132)* suppressor mutations that map to Chromosome I, so this chromosome is not exhausted as a source of new spermatogenesis genes. Further, spermatogenesis is a process that requires an inordinately large percentage of the genome. Well over 5% of the genome is enriched or expressed solely during spermatogenesis [44] [30], which probably reflects the unique nature of the sperm cell. However, fewer than 100 genes that have been ascribed a sperm function through experimental studies. Therefore, we understand only a small fraction of the mechanistic processes governing spermatogenesis; many more await investigation.

## Methods

### Worm Strains and Culture

All *C. elegans* strains were maintained on *Escherichia coli* OP50-seeded Nematode Growth Media (NGM) agar plates and manipulated as described by Brenner [45]. The *Caenorhabditis* Genetic Center kindly provided the following strains: N2, CB4856, *spe-27(it132ts)IV, spe-27(it132ts) unc-22(e66)IV, spe-29(it129) dpy-20(e1282ts)IV, him-5(e1490)V, spe-27(it132ts)IV; him-5(e1490)V, fem-1(hc17ts)IV, fem-3(q23ts)IV, rrf-3(pk1426)II, eri-1(mg366 IV* and *mnDf111/unc-13(e1091) lin-11(n566)I*. The strain bearing the *hc197* mutation was isolated in a suppressor screen of *spe-27(it132ts); unc-22(e66)* [17] [15]. We backcrossed the *hc197; spe-27(it132ts) unc-22(e66)* line six times with *spe-27(it132ts),* each time recovering *unc-22* F2 that were fertile at 25°C. Worms were mated in 35 mm petri dishes at a ratio of 12 males to four hermaphrodites unless otherwise noted. In creating the *spe-46(hc197)/mnDf111* hemizygotes, *spe-46(hc197)/+; him-5* males were crossed to *mnDf111/unc-13(e1091) lin-11(n566)* hermaphrodites. The F1 were allowed to lay eggs for three days, after which their DNA was extracted, the region containing the *hc197* mutation was amplified by PCR, and the DNA was sequenced to confirm the genotype.

### Single Nucleotide Polymorphism Mapping of hc197

The *hc197* mutation was mapped using its *spe-27(it132ts)* suppression phenotype. We constructed a mapping strain which had *spe-27(it132ts)* backcrossed into the Hawaiian strain CB4856 six times (henceforth HA-*spe-27*). In the mapping cross, *hc197; spe-27(it132ts) unc-22(e66)* hermaphrodites were mated to HA-*spe-27* males. Virgin F1 progeny were allowed to self-fertilize at 15°C, where *spe-27(it132ts)* worms are fertile. A total of 120 F2 worms were isolated as L4 larvae and allowed to reproduce at 25°C, where only *hc197* homozygotes are self-fertile in a *spe-27* background. Twenty-seven of the F2 were identified as fertile. These fertile worms were combined for bulk DNA extraction, as were another 27 sterile F2 worms chosen arbitrarily, essentially as described [31]. The results from this approach to SNP mapping are in the form of map ratios, where ratios near 1.0 indicate an unlinked state, and map ratios close to 0.0 indicate linkage. We initially tested the following SNP loci for linkage to *hc197* (Chromosome: nucleotide position): pKP1119 (Chr I), pkP2107 (Chr II), pkP3103 (Chr III), pkP5097 (Chr V) (see Fig. 1 for chromosomal locations). We did not analyze SNPs on chromosome IV because preliminary data indicated *hc197* was unlinked to *spe-27* on chromosome IV. Only the Chromosome I SNP had a map ratio indicating linkage (Fig. 1), so we subsequently analyzed three more SNP loci across Chromosome I (Fig. 1). All SNP information is based upon WormBase release WS230.

### Sequencing and Analysis of the *hc197* Genome

Genomic DNA was extracted from ∼1 ml of worms from the *hc197; spe-27(it132ts) unc-22(e66)* strain using standard methods. Briefly, we froze ∼0.5 ml of worms in 4 ml of TEN (20mM Tris, 50mM EDTA, 100mM NaCl) and digested them for 0.5 hours at 60°C in TEN plus 0.5% SDS and 0.1 mg/ml Proteinase K. The concentration of Proteinase K was increased to 0.2 mg/ml, and the digestion was extended for another hour. DNA was extracted from the digest via phenol/chloroform/isoamyl alcohol and precipitated with NaOAc/EtOH. The pellet was resuspended in TEN, RNase A was added to 40 mg/ml, and the digestion was incubated for an hour. The DNA was extracted and precipitated as above and resuspended in TE. DNA sequencing was conducted at the City of Hope and Beckman Research Institute’s DNA Sequencing/Solexa Core (Duarte, CA), where sequencing with the Illumina Genome Analyzer II platform was performed on two flow cell lanes with 80 bp paired-end reads from the ends of ∼350 bp fragments of the DNA, generating a total of 82,301,352 reads. Using the bioinformatics software Geneious Pro™ (version 5.6.2) 14.2% of the reads, or 11,687,711 reads, were mapped to Chromosome I (Accession no. NC_003279). This percentage corresponds closely to the percentage of the genome found on Chromosome I (15.8%) and provided an average depth of 62 reads per base pair.

### Microinjection Transformation

Worms were transformed with PCR products from one of the two genes tested plus flanking sequence (W06D4.2 plus 1,545 bp upstream and 732 bp downstream; T24B1.1 plus 1,324 bp upstream and 677 bp downstream). The sequences were amplified from N2 DNA using the Expand High Fidelity^PLUS^ PCR System (Roche Diagnostics) following the manufacturer’s protocols. The amplicons were purified using the Wizard® SV Gel and PCR Clean-Up System (Promega). An injection mix of the PCR product (15 ng/µl) along with a plasmid containing *myo-3*::mCherry (pCFJ104, 100 ng/µl) was microinjected into the gonads of young adult hermaphrodites. Transformed worms were identified by mCherry fluorescence in the body wall muscle. F2 transformants were scored for rescue, but transformed lines were not maintained past the rescue experiments because transgenes expressed in sperm are generally rendered ineffective by germline silencing after a small number of generations [46].

### RT-PCR

RNA was extracted from mixed age populations of worms from the strains *fem-1(hc17ts), fem-3(q23ts), spe-46(hc197),* and N2. Large populations of each strain were collected and rinsed 4 times with M9 buffer. After freezing at -80°C, the worms were disrupted by sonication in TRIzol® Reagent, and the RNA was extracted following the manufacturer’s protocols (Life Technologies™). RNA samples were treated with RQ1 DNase (Promega) and subsequently purified via phenol-chloroform isoamyl alcohol extraction, and samples were diluted to 33 ng/µl. RT-PCR was performed with the MyTaq™ One-Step RT-PCR Kit (Bioline) according to manufacturer’s instructions. We multiplexed *spe-46* specific primers (Exon2F: 5′-GGCAGAGAATAGTCCATTGG-3′; and Exon5R: 5′-TTGCTTGACTCTCTCCAACG-3′) with primers specific to *act-2,* the *C. elegans* homolog of β-Actin (actinF: 5′-GTATGGGACAGAAAGACTCG-3′; and actinR: 5′-CGTCGTATTCTTGCTTGGAG-3′).

### Construction of a *spe-46* RNAi Clone and Induction of RNA Interference

Using the MyTaq™ One-Step RT-PCR Kit a product was amplified from N2 RNA using primer pairs RNAiF and RNAiR (5′-gtgtGCGGCCGCgtgtcattgcatattacgatgg-3′ and 5′- gtgtACTAGTaagtggtaatgaagacgctg-3′, respectively). Primers were engineered with an added restriction site on the 5′ end for cloning: NotI on the forward primer and SpeI on the reverse primer (uppercase letters correspond to restriction enzyme sites; the extreme 5′-gtgt was added to each primer to maximize restriction enzyme activity). The amplicon was cloned into the L4440 RNAi vector [47], and transformed into *E. coli* HT115(DE3), a strain which lacks RNase III activity. Expression of dsRNA from the insert was achieved through isopropyl-**β**-D-thiogalactopyranoside (IPTG) induction of T7 promoter sites flanking the L4440 multiple cloning site.

Worms were exposed to the RNAi feeding strain bacteria in petri dishes on agar prepared with 25 µg/ml carbenicillin and 1 mM IPTG. Plates were seeded with transformed HT115 bacteria that had been grown the previous night in a 2 ml liquid culture with LB media, 50 µg/ml carbenicillin, and 15 µg/ml tetracycline. In the final hour of liquid culture, IPTG was added to a final concentration of 1 mM. Large populations of worms were bleached [48] to obtain eggs, which were introduced onto plates. At the L4 larval stage, exposed hermaphrodites were transferred to their own RNAi plate, and their lifetime fecundity was measured. Identically handled control worms were exposed to HT115 bacteria containing the L4440 empty vector.

### Construction of a *spe-46::gfp* Reporter

A transcriptional *spe-46::gfp* construct was integrated into Chromosome II following the Mos-SCI technique [49]. Initially, the spe-46 promoter was amplified from N2 DNA, as was the spe-46 3′ UTR. The promoter and 3′ UTR were stitched together with the GFP coding sequence following the PCR fusion technique described by Hobert [50]. The promoter-GFP-3′ UTR fusion was cloned into the multiple cloning site of the vector pCFJ151; the final construct contained 1,015 bp upstream of the spe-46 start codon (the next gene begins 846 bp upstream on the opposite strand), followed by the GFP coding sequence, and finally 1,020 bp downstream of the spe-46 stop codon. The pCFJ151 vector targets the ttTi5605 mos1 insertion on Chromosome II for homologous recombination, and we recovered a homozygous integrated copy of the construct [49].

### Microscopy, *in vitro* Spermatid Activation, and Microinjection Transformation

Imaging was accomplished on a Nikon Eclipse Ti inverted microscope outfitted for Nomarski DIC and epifluorescence. Nuclear material was labeled with 30 ng/µl Hoechst 33342 (Life Technologies™) in SM1 buffer [51]. Fused MOs were visualized with SM1 containing 2µM FM® 1–43 (Life Technologies™), a vital fluorescent dye that labels the outer leaflet of plasma membrane [14]. On the other hand, all MOs were visualized with an Alexa Fluor® 594 conjugate of wheat germ agglutinin (WGA; Life Technologies™). Here, the sperm were fixed in 4% paraformaldehyde in SM1, rinsed 3 times in PBS, permeabilized with 0.5% Triton X-100 in PBS, rinsed another 3 times with PBS, and labeled with 5 µg/ml of WGA in PBS. In other experiments, sperm were activated *in-vitro* by exposure to SM1 containing either 200 µg/ml Pronase or 70 mM TEA (pH 7.8). Images were captured on a Nikon DS-Qi1 12 bit monochrome camera and analyzed with Nikon NIS Elements software. The same microscope system was used for microinjection of DNA into the gonads of recipient young adult hermaphrodites. The injection mix contained 10–15 ng/µl of the transgenes for rescue and 100 ng/µl of the transformation marker.
